# Coordinate transcriptional regulation of ErbB2/3 by C-terminal binding protein 2 signals sensitivity to ErbB2 inhibition in pancreatic adenocarcinoma

**DOI:** 10.1038/s41389-023-00498-8

**Published:** 2023-11-10

**Authors:** Kranthi Kumar Chougoni, Haemin Park, Priyadarshan K. Damle, Travis Mason, Bo Cheng, Martin M. Dcona, Barbara Szomju, Mikhail G. Dozmorov, Michael O. Idowu, Steven R. Grossman

**Affiliations:** 1 Keck School of Medicine and USC Norris Comprehensive Cancer Center, University of Southern California, Los Angeles, CA 90033 USA; 2 Department of Internal Medicine, Virginia Commonwealth University, Richmond, VA 23298 USA; 3 Department of Surgery, Virginia Commonwealth University, Richmond, VA 23298 USA; 4 Department of Biostatistics, Virginia Commonwealth University, Richmond, VA 23298 USA; 5 VCU Massey Comprehensive Cancer Center, Virginia Commonwealth University, Richmond, VA 23298 USA; 6 Department of Pathology, Virginia Commonwealth University, Richmond, VA 23298 USA

**Keywords:** Pancreatic cancer, Tumour biomarkers, Cancer models, Targeted therapies

## Abstract

There is a critical need to identify new therapeutic vulnerabilities in pancreatic ductal adenocarcinoma (PDAC). Transcriptional co-regulators C-terminal binding proteins (CtBP) 1 and 2 are highly overexpressed in human PDAC, and CRISPR-based homozygous deletion of *Ctbp2* in a mouse PDAC cell line (CKP) dramatically decreased tumor growth, reduced metastasis, and prolonged survival in orthotopic mouse allografts. Transcriptomic profiling of tumors derived from CKP vs. *Ctbp2*-deleted CKP cells (CKP/KO) revealed significant downregulation of the EGFR-superfamily receptor Erbb3, the heterodimeric signaling partner for both EGFR and ErbB2. Compared with CKP cells, CKP/KO cells also demonstrated reduced Erbb2 expression and did not activate downstream Akt signaling after stimulation of Erbb3 by its ligand neuregulin-1. ErbB3 expression in human PDAC cell lines was similarly dependent on CtBP2 and depletion of ErbB3 in a human PDAC cell line severely attenuated growth, demonstrating the critical role of ErbB3 signaling in maintaining PDAC cell growth. Sensitivity to the ErbB2-targeted tyrosine kinase inhibitor lapatinib, but not the EGFR-targeted agent erlotinib, varied in proportion to the level of ErbB3 expression in mouse and human PDAC cells, suggesting that an ErBb2 inhibitor can effectively leverage CtBP2-driven transcriptional activation of physiologic ErbB2/3 expression and signaling in PDAC cells for therapeutic benefit.

## Introduction

Pancreatic adenocarcinoma (PDAC) is among the most lethal of cancers, with an average 5-year survival from diagnosis of only 10% [1]. Only limited success is observed with existing therapies, and there is an immediate need to fully understand the underlying pathogenesis of PDAC to identify novel and effective therapies. We have previously demonstrated that the oncogenic transcription factor CtBP2 [2], which is frequently overexpressed in pancreatic cancer, as well as colon, breast, ovarian, prostate, and gastric cancers and associated with poor patient outcomes [3–6], is a critical dependency in PDAC, as *Ctbp2* allelic loss led to dramatic decreases in tumor size and metastasis along with prolonged survival in PDAC-prone CKP (*Pdx1-Cre; LSL-Kras*^*G12D/+*^*; Trp53*^*fl/fl*^) mice [4]. Although the decreased tumor size and metastasis and prolonged survival seen in *Ctbp2-*deficient CKP mice were accompanied by reduced expression of cancer stem cell markers, the exact mechanism for dependency of PDAC tumor progression on Ctbp2 has remained unclear [4].

In this report, we have used a cell line derived from a CKP mouse PDAC tumor in which we homozygously deleted the *Ctbp2* gene via CRISPR, to further interrogate the oncogenic mechanism of action of Ctbp2 in PDAC progression using an orthotopic allograft PDAC mouse model. Indeed, deletion of *Ctbp2* significantly decreased PDAC tumor growth and metastasis and prolonged survival in mice orthotopically implanted with *Ctbp2*-deficient CKP cells, compared with mice implanted with parental CKP cells. Transcriptional profiling of CKP and *Ctbp2*-deficient CKP orthotopic PDAC tumors identified a novel connection between *Ctbp2* and *Erbb3* expression, also seen in human PDAC cell lines, and we further demonstrated Ctbp2-dependence for signaling by Erbb2/Erbb3 complexes after stimulation with the ErbB3 ligand neuregulin-1 (NRG-1). Depletion of ErbB3 in a human PDAC cell line strikingly curtailed growth, suggesting that ErbB3 and its physiologic signaling partner ErbB2, might serve as therapeutic targets in ErbB3-expressing PDAC. Indeed, mouse and human PDAC cells with higher levels of *ErbB3* expression were significantly more sensitive to the ErbB2-targeted tyrosine kinase inhibitor (TKI) lapatinib. Taken together, our studies reveal novel regulation of ErbB3 by CtBP2 in PDAC, opening new avenues to target ErbB3-expressing PDAC tumors with inhibitors of EGFR superfamily signaling.

## Results and discussion

### Ctbp2 drives PDAC tumor growth and metastasis in an orthotopic allograft mouse model

**Fig. 1:**
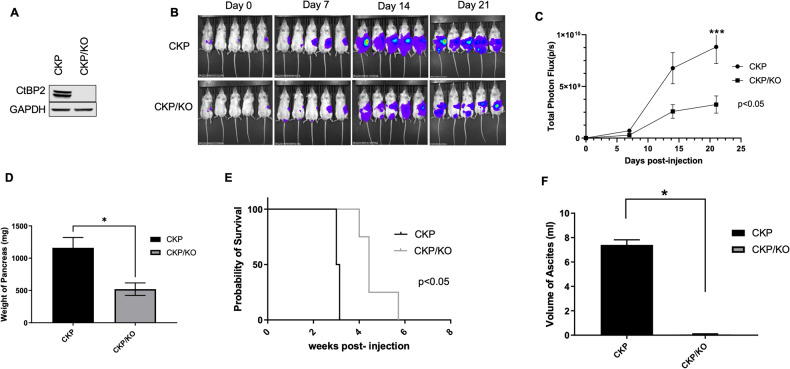
Ctbp2 drives tumor growth and metastasis in an orthotopic PDAC allograft mouse model. **A** Lysates of parental CKP and CKP cells with homozygous CRISPR/Cas9-based deletion of *Ctbp2* (CKP/KO) immunoblotted with anti-Ctbp2 and GAPDH (loading control) antibodies. **B** Bioluminescent images obtained on the indicated days after orthotopic pancreatic implantation of luciferase-expressing CKP-luc or CKP/KO-luc cells into NSG mice. **C** Quantification of mean photon flux per second corresponding to images of mice in (**B**) using Living Image software (Perkin Elmer). **D** Total weight of pancreata of the mice in (**B**) at necropsy on day 21. **E** Kaplan–Meier survival analysis of mice with orthotopically implanted CKP-luc or CKP/KO-luc cells that were observed until humane endpoint. **F** Ascitic fluid volume of the mice in (**B**) at necropsy on day 21. (*n* = 5/group). *p < 0.05 and ***p < 0.001. *p* Values were obtained by performing two-tailed paired *t*-test between the two groups. Error bars indicate ±1.0 SD. *Methods:* (CKP PDAC cell line) The mouse CKP PDAC cell line was developed from the pancreas of a two-month-old CKP mouse according to the procedure described in [7]. The CKP cell line was verified as derived from PDAC tumor cells by showing elevated expression of PDAC markers *Muc4* and *Krt19* in the CKP cells vs. cells derived from normal mouse pancreata (Fig. S1B). CKP and all derivative cell lines were confirmed mycoplasma-free using a PCR-based assay (Cat No: ab289834; Abcam, Waltham, MA, USA). (CRISPR/Cas9-deletion of *Ctbp2*) We employed CRISPR-Cas9 gene editing to knock out *Ctbp2* in CKP cells, and the resulting clones were validated for deletion of *Ctbp2* by PCR of the *Ctbp2* chromosomal locus and Ctbp2 immunoblotting (see Fig. 3 Methods). One of the several clones generated (CKP/KO) was used for all subsequent experiments. CKP and CKP/KO cell lines were grown in monolayers using RPMI media (Thermo Fisher, Waltham, MA, USA) supplemented with 4% FBS (Corning Life Sciences, Durham, NC, USA) and 1% penicillin–streptomycin (Thermo Fisher) at 37 °C in 5% CO_2_. DNA encoding CRISPR sgRNA targeting exon 3 of *mCtbp2* was cloned into CRISPR OFP Nuclease Vector (Thermo-Fisher) using DNA oligonucleotides CP01 and CP02 (Table S2). *mCtbp2* CRISPR plasmid-transfected CKP cells were sorted by flow cytometry 72 h post-transfection (*λ*_exc_ = 488 nm). Cells with highest Orange Fluorescent Protein (OFP) expression were pooled and cultured for a week until OFP expression was completely diminished. These cells were re-sorted for absence of OFP, single cell seeded in 96 well plates and grown to confluence. The clones were replica-plated for screening by western blotting. Further confirmation of homozygous knockout was done by PCR-amplification of the flanking regions of the indels with oligonucleotides DP331 and DP332 (Table S2) followed by cloning into pGEM-T Vector (Promega, Madison, WI, USA). Clones were sequenced to identify mutations in the *mCtbp2* allele. (Luciferase expression in CKP and CKP/KO cells) The lentiviral vector pFULT expressing luciferase linked to Tomato under the control of the human pPGK1 promoter was purchased from Northwestern University (Gene Editing Transduction & Nanotechnology Core). CKP and CKP/KO cells were infected with this virus and 24 h later, the media in the cell culture dish was replaced with fresh media. After 24 h, lentiviral-infected cells were selected with puromycin (1.25 µg/ml) for 7 days, after which we performed an in vitro luciferase assay to confirm luciferase expression. All cells in the control plate died due to puromycin treatment. (Orthotopic injection of PDAC cells) We injected approximately 8 × 10^5^ luciferase-expressing CKP-luc and CKP/KO-luc cells in PBS mixed with an equal volume of Cultrex Basement Membrane Matrix type 3 (R&D Systems, Minneapolis, MN, USA) into the pancreatic tail of 4-month-old male NSG mice (5 mice/cell line) as described [26–28]. NSG mice were procured from the Virginia Commonwealth University (VCU) Cancer Mouse Models Core Laboratory, and all animal studies were approved by the VCU Institutional Animal Care and Use Committee. The adherence of the implanted cells to the pancreas was measured immediately after orthotopic implantation by bioluminescent imaging in the IVIS Spectrum imager (Perkin-Elmer, Waltham, MA, USA) performed 10 min after subcutaneous injection of D-luciferin (150 mg/kg). Tumor growth was monitored similarly on days 7, 14, and 21 after implantation, via bioluminescent imaging after injection of the mice with D-luciferin.

To fully understand the role of CtBP2 in PDAC tumor biology, we developed a tractable orthotopic allograft model utilizing a mouse PDAC cell line derived from PDAC tumors of CKP mice [4] (Fig. S1A). The cell line (CKP) was validated as of ductal epithelial origin, as we observed higher mRNA expression of the epithelial/PDAC markers *Krt19* (CK19) and *Muc4* [7] in CKP cells compared to normal mouse pancreas tissue (Fig. S1B). *Ctbp2* was then deleted homozygously in the CKP cell line using CRISPR/Cas9, with the *Ctbp2*-deleted CKP/KO cells demonstrating complete loss of Ctbp2 protein expression as confirmed by Ctbp2 immunoblot (Fig. 1A). To facilitate in situ imaging, the CKP and CKP/KO cell lines were next transduced with a luciferase cDNA, and luciferase expression in both cell lines (CKP-luc and CKP/KO-luc) was confirmed as equivalent by in vitro luciferase assay (Fig. S1C).

To better understand any differences between the phenotype of CKP and CKP/KO cells in vivo, we first determined whether Ctbp2 was required for growth in cell culture. Though both CKP-luc and CKP/KO-luc cell lines grew similarly over the initial 5 days of the assay, there was a modest but statistically significant 30% decrease in final live cell number achieved after 8 days of growth for the CKP/KO cells (Fig. S2A). To investigate the putative in vivo role of Ctbp2 in PDAC tumor growth and metastasis, CKP-luc and CKP/KO-luc cell lines were injected orthotopically into the pancreatic tail of 4-month-old immune-deficient non-obese diabetic (NOD)-SCID γ (NSG) mice, which were chosen so as to negate any impact of the host mouse immune system, allowing focus on the cell autonomous effects of *Ctbp2*. Mice were monitored for tumor growth over 3 weeks or until a humane endpoint was reached. Tumors in mice implanted with CKP/KO-luc cells grew significantly more slowly than CKP-luc tumors with final photon flux ~1/3 of that seen in CKP-luc tumors as determined by interval luciferase bioluminescent imaging (Fig. 1B, C). At necropsy, the average weight of pancreata from the CKP cohort was also 2-fold higher than the CKP/KO group, reflecting a higher overall tumor burden in the pancreas (Fig. 1D). Consistent with smaller tumor size, mice implanted with CKP/KO-luc cells survived significantly longer than mice implanted with CKP-luc cells, with calculated median survivals of 4.4 vs. 3 weeks, respectively (Fig. 1E).

We found a significant increase in the body weight of the mice carrying CKP tumors as compared to CKP/KO tumors (Fig. S2B), that was likely due to accumulation of ascitic fluid (a clear indicator of peritoneal metastasis in human PDAC [8]) which was present in all CKP mice, but in none of the CKP/KO mice (Fig. 1F). These findings therefore suggested that Ctbp2 might be crucial not only for PDAC growth, but also metastasis. Consistent with the universal finding of ascites in the CKP mice, mice implanted with CKP cells also demonstrated extensive peritoneal metastases (peritoneal surfaces of the liver, kidney, spleen, intestines), while only 1 of 5 CKP/KO injected mice exhibited peritoneal metastases, and only to the surfaces of the liver and spleen in the one mouse with metastasis (Fig. S2C and S2D; Table S1). This finding correlates with our previous observations in the CKP mouse model, where allelic loss of *Ctbp2* eliminated metastasis to the peritoneum, the preferred site of metastasis in transgenic mice born with the CKP genotype [4].

We further investigated the underlying mechanism for the differential metastatic potential of CKP vs. CKP/KO tumors by studying the migratory potential of CKP vs. CKP/KO cells in vitro using a trans-well migration assay. Based on our previous data in colon cancer cells [9], we expected that CKP/KO cells would exhibit a deficiency in migratory ability and indeed, we observed a decrease in the ability of CKP/KO cells to migrate in this assay when compared with CKP cells (Fig. S2E). Thus, Ctbp2 directs both the growth and metastatic potential of PDAC tumor cells, with metastatic phenotypes in vivo correlating with activation of a migratory phenotype in vitro.

### CtBP2 regulates expression of ErbB3, which is critically required for PDAC cell growth

**Fig. 2:**
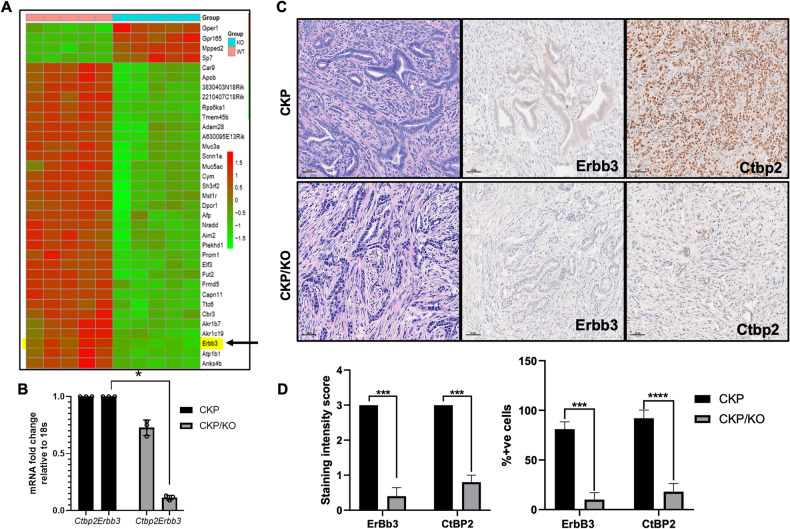
Ctbp2 drives Erbb3 expression in orthotopic mouse PDAC allografts. **A** Heatmap showing the top 35 differentially regulated genes between CKP (“WT”) and CKP/KO (“KO”) tumors from NSG mice 3 weeks after orthotopic injection of cells (*n* = 5/group). **B** Validation of RNAseq results by qPCR. The relative mRNA levels of the indicated genes were measured by qPCR using SYBR Green probes and the 2^−ΔΔCt^ method (*n* = 3 independent biological replicates/ group). **C** Representative CKP and CKP/KO PDAC tumors from orthotopically implanted NSG mice stained by IHC for Erbb3 and Ctbp2. Scale bar indicates 50 µM. **D** (left) Mean Erbb3 and Ctbp2 staining intensity (scale 0–3; [29]) and (right) mean percent positive cells staining for Ctbp2 and Erbb3 in CKP and CKP/KO tumors. Five random fields were analyzed from each tumor. (*n* = 5 independent tumors). *p < 0.05 and ***p < 0.001. *p* Values were obtained by performing two-tailed paired *t*-test between the two groups. Error bars indicate ±1.0 SD. *Methods:* (RNAseq analysis of CKP vs. CKP/KO tumor mRNA) Immediately after euthanizing mice on day 21 after orthotopic implantation of CKP or CKP/KO cells as described in Fig. 1B, pancreata were harvested under ice-cold conditions. The isolated tissues were powdered by trituration with liquid nitrogen, and RNA was extracted with Triazole (Triazole LS; Ambion, Carlsbad, CA, USA) and chloroform (MilliporeSigma, Burlington, MA, USA) as described [30]. Total RNA was purified using RNeasy Mini kit (Qiagen) according to the manufacturer’s recommendations. RNA integrity number (RIN) value was assessed on an Agilent 2100 Bioanalyzer (Agilent, Santa Clara, CA, USA), and samples with RIN values above 9 were selected for sequencing purposes. Prior to performing RNA-Seq, ribosomal RNA was depleted from the samples using the RiboMinus Human/Mouse Transcriptome Isolation Kit (Thermo Fisher). All samples were sequenced (University of Texas, Southwestern Medical Center- Next Generation Sequencing Core Lab) and ~29 million 50 bp single-end reads per sample were obtained. Sequencing adapters were removed using Trimmomatic v.0.33 [27]. Quality control at each processing step was performed using the FastQC tool v0.11.2 (quality base calls, CG content distribution, duplicate levels, complexity level). The Mouse GRCm38/mm10 reference genome was obtained from the UCSC Genome Browser Gateway (http://hgdownload.soe.ucsc.edu/goldenPath/mm10/bigZips/chromFa.tar.gz), and the corresponding gene annotation file was obtained from Ensemble (ftp://ftp.ensembl.org/pub/release-83/gtf/mus_musculus/Mus_musculus.GRCm38.83.gtf.gz). Reads were aligned using the subread v.1.6.2 aligner. We obtained gene counts using the featureCounts v.1.2.6 software [28]. RNA-seq counts were preprocessed and analyzed for differential expression using edgeR v.3.24.3 [29]. *p*-Values for differentially expressed genes were corrected using a false discovery rate (FDR) multiple testing correction method [30]. Functional enrichment analysis (GO, KEGG) was performed using enrichr [31]. Enrichment analysis using custom signatures was performed using a hypergeometric test in the clusterProfiler v.3.10.1 R package [32]. Row-median centered log_2_(T P M + 1) expression profiles for selected genes were visualized using the pheatmap package v.1.0.12. All statistical calculations were performed within the R/Bioconductor environment v3.5.3. The raw and processed data are available on GEO (https://www.ncbi.nlm.nih.gov/geo/query/acc.cgi?&acc=GSE228625). (qPCR of tumor mRNA) cDNA from extracted RNA was prepared using SensiFAST cDNA Synthesis Kit (Meridian Bioscience, Cincinnati, OH, USA) as per the manufacturer specifications. The mouse qPCR primers were designed using Primer Bank (https://pga.mgh.harvard.edu/primerbank/) and validated using the NCBI BLAST program. qPCR was carried out using SYBR Green probes. Relative mRNA expression was calculated by 2^−^^ΔΔCt^ method using 18S rRNA as internal standard using primer pairs described (Table S3). (**IHC**) Paraffin embedded tissue sections were deparaffinized by washing the slides in Citra Solv (Decon Labs, King of Prussia, PA, USA) and then hydrated by washing in a series of graded ethanol concentrations (2× each for 5 min in 100%, 90% and 1× each for 5 min in 80 and 70%), following which antigen unmasking was performed by heating the tissue sections in retrieving solution (Retrievagen A, pH 6.0; BD Biosciences, Franklin Lakes, NJ, USA) using Retriever 2100 (Aptum Biologics, Southampton, UK) as per manufacturer instructions. Slides were next incubated in 3% hydrogen peroxide to quench endogenous peroxidase activity and tissue sections were circled using a hydrophobic PAP pen. After washing in 10 mM Na_2_HPO_4_, 137 mM NaCl, 2.7 mM KCl, 1.8 mM KH_2_PO_4_, 0.1 % Tween 20 pH 7.4 (PBST), the slides were incubated in blocking buffer (5% goat serum in PBST) for 1 h at room temperature. After three 5-minute washes in PBST, the slides were incubated with the following primary antibodies: CtBP2 (Cat No: sc-5966, Goat anti-CtBP2; 1:100; Santa Cruz Biotechnologies, Dallas, TX, USA), ErbB3 (Cat No: 12708, Rabbit anti-ErbB3; 1:200; Cell Signaling Technology, Danvers, MA, USA) overnight at 4 °C. After a series of washes in PBST, slides were incubated with HRP-conjugated anti-rabbit (Cat No: 111-035-003, 1:500; Jackson ImmunoResearch, West Grove, PA, USA) and anti-goat (Cat No: sc-2354, 1:200; Santa Cruz Biotechnologies) secondary antibodies for 1 h at room temperature. After 3 further washes in PBST, the slides were stained with diaminobenzidine (DAB) per manufacturers protocol (DAKO Chromogen, Agilent) and counter-stained using Mayer’s Hematoxylin for 5 min. Slides were dehydrated by washing through graded alcohols (70%, 80%, 95% 1× each for 3 min and 100% 2× each for 5 min), then 3× for 5 min each in Citra Solv [31], and cover slipped using mounting media (Permount, Electron Microscopy Sciences, Hatfield, PA, USA). IHC scoring was performed by a blinded pathologist as described [29].

To better understand molecular pathways contributing to CtBP’s oncogenic activities in the orthotopic CKP PDAC model, we performed RNA sequencing (RNA-seq) analysis to compare differentially expressed genes between CKP and CKP/KO tumors (*n* = 5/group; false discovery rate = 0.01; Fig. 2A). A total of 780 protein coding genes were altered in response to deletion of *Ctbp2*, and of the top fifty genes that were differentially expressed between the two groups, we noted significant downregulation of the EGFR superfamily member *Erbb3*, which is a biochemical and functional partner to both the ErbB1/EGFR (EGFR hereafter) and ErbB2/Her2 (ErbB2 hereafter) proto-oncoproteins [10]. This finding was validated by qPCR analysis which demonstrated a striking 80% reduction in *Erbb3* mRNA in CKP/KO compared with CKP tumors (Fig. 2B).

To investigate whether Erbb3 regulation by Ctbp2 was specifically occurring within CKP mouse tumor cells, as opposed to adjoining tumor stroma or normal tissue, we performed Ctbp2 and Erbb3 immunohistochemical (IHC) staining of pancreatic tumors from mice orthotopically implanted with CKP or CKP/KO cells, scoring mean staining intensity and the percentage of positively staining tumor cells for each marker across multiple tumors from each cell type (*n* = 5) (Fig. 2C, D). As expected, Ctbp2 and Erbb3 were both strongly expressed (mean intensityes = 3.0 for both) and present in nearly all (90 and 80% positive, respectively) CKP tumor cells, while the mean staining intensity of Ctbp2 and Erbb3 was minimal in tumors derived from CKP/KO tumor cells (0.8 and 0.4, respectively) with only a minority of tumor cells demonstrating any detectable expression (20 and 10% positive, respectively) (Fig. 2C, D). These data support direct and tumor cell-autonomous regulation of Erbb3 expression by Ctbp2 in CKP PDAC tumor cells.

**Fig. 3:**
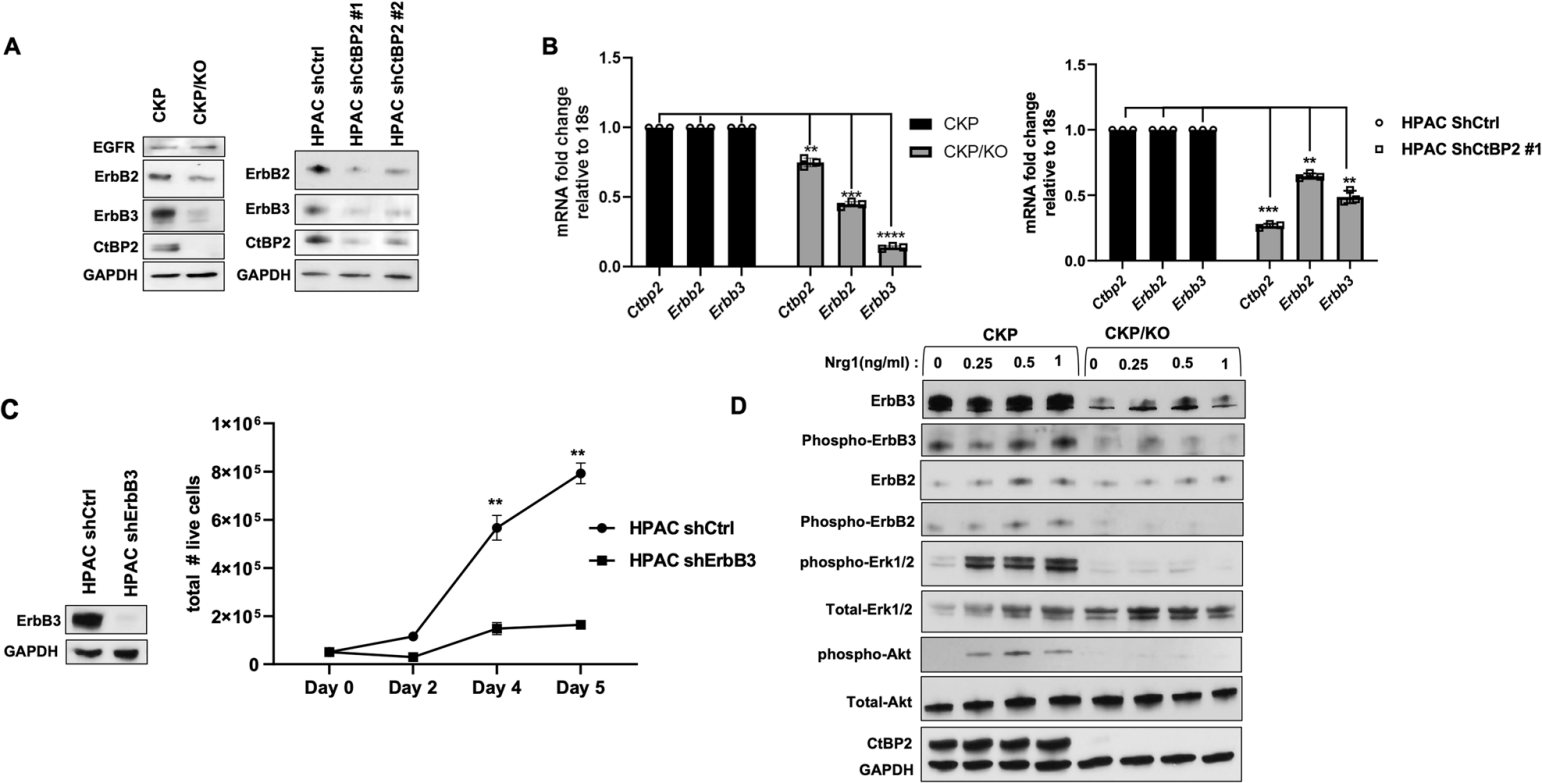
A CtBP2/ErbB2/3 axis regulates Akt/MAPK signaling and growth potential of PDAC cells. **A** Immunoblot analysis of EGFR, ErbB2, ErbB3, CtBP2, and GAPDH (loading control) in lysates of CKP vs. CKP/KO and HPAC cells expressing control (shCtrl) or CtBP2-targeted (shCtBP2) shRNAs. **B** *ErbB2/3* and *CtBP2* mRNA levels were estimated by qPCR in CKP vs. CKP/KO and HPAC shCtrl vs. shCtBP2 cells. **C** (left panel) Lysates of shCtrl or shErbB3-expressing HPAC cells were immunoblotted with ErbB3 and GAPDH (loading control) antibodies. (right panel) The growth of shCtrl or shErbB3-expressing HPAC cells was monitored over 5 days using trypan blue staining to detect live cells as described [32]. **D** Serum-starved CKP vs. CKP/KO cells were exposed to the indicated concentrations of recombinant mouse NRG-1 for 15 min and cell lysates were immunoblotted with the indicated antibodies. GAPDH was a loading control. *n* = 3 independent experiments. **p < 0.01, ***p < 0.001 and ****p < 0.0001. *p* Values were obtained by performing two-tailed paired *t*-test between the two groups. Error bars indicate ±1.0 SD. *Methods:* (Generation of shRNA-expressing HPAC cell lines) HPAC (ATCC, Manassas, VA, USA) and all other human cells used in Fig. 4 were grown in monolayers using DMEM media (Thermo Fisher) supplemented with 10% FBS (Corning) and 1% penicillin–streptomycin (Thermo Fisher) at 37 °C in 5% CO_2_. HPAC cell lines stably expressing control, CtBP2, or ErbB3-targeted shRNAs were derived by transduction of control, shCtBP2, or shErbB3-expressing lentiviruses prepared using the plasmids pLKO1-shCtrl (Cat No: SHC016; Sigma, St. Louis, MO, USA), pLKO.1-shCtBP2 (Sigma), and pLKO.1-ShErbB3 (Sigma) as described [33], followed by selection using puromycin (2 µg/ml) and verification of knockdown of intended shRNA targets by immunoblotting. (NRG-1 stimulation) CKP and CKP/KO cells were serum-starved overnight after reaching 80% confluency and treated with recombinant mouse NRG-1 (R&D Systems) at indicated concentrations for 15 min. Cells were then washed with ice-cold PBS, scraped on ice, and pelleted by cold centrifugation at 8000 rpm for 5 min at 4 °C. The cell pellet was lysed using cold 1× RIPA buffer (Cell Signaling Technology) containing protease and phosphatase inhibitors (Mini EDTA tablet, Roche) freshly added on ice and incubated for 10 min. Lysates were then centrifuged at 12,000 rpm for 10 min at 4 °C, protein concentration in the lysate was measured using BCA reagent (Thermo Fisher), and 30 µg of total protein was mixed with LDS buffer [1×] and heated for 10 min at 95 °C followed by immunoblotting. (Immunoblotting) Primary antibodies used for immunoblotting included: CtBP2 (Cat No: 612044, 1:1000; BD Biosciences), GAPDH (Cat No: AB 2302, 1:20000; MilliporeSigma), EGFR (Cat No: sc-03, 1:500; Santa Cruz Biotechnologies), Phospho-ErbB3 (Cat No: 4791, 1:1000; Cell Signaling Technology), ErbB3 (Cat No: 12708, 1:000; Cell Signaling Technology), Phospho-ErbB2 (Cat No: 2243, 1:1000; Cell Signaling Technology), ErbB2 (Cat No: sc-33684, 1:500; Santa Cruz Biotechnology), Total-Akt (Cat No: 9272, 1:1000; Cell Signaling Technology), Phospho-Akt (Cat No: 3787, 1:1000; Cell Signaling Technology), Total Erk1/2 (Cat No: 9102, 1:1000; Cell Signaling Technology), Phospho-Erk1/2 (Cat No: 4370, 1:1000; Cell Signaling Technology). After incubation in HRP-conjugated anti-rabbit or anti-mouse secondary antibodies (Cat Nos: 1706515 and 1706516, 1:10,000; Bio-Rad, Hercules, CA, USA), membranes were detected by chemiluminescence (Western Lightning Pro, Perkin-Elmer).

To determine if Ctbp2 regulation of Erbb3 in mouse tumors was generalizable to human PDAC, we examined a panel of 9 human PDAC cell lines for CtBP1/2 and ErbB3 protein expression, and while only 5 of the 9 lines expressed detectable ErbB3, all cell lines expressed both CtBP1 and 2 equivalently (Fig. S3). To validate CtBP2 control of ErbB3 expression in human PDAC cells as seen in mouse CKP cells, we expressed control or CtBP2 shRNA in the ErbB3-positive human PDAC cell line HPAC, and as observed in CKP/KO cells, there was a drastic reduction in *ErbB3* mRNA and protein levels after CtBP2 depletion (Fig. 3A, B).

### Active ErbB2/ErbB3/Akt/MAPK signaling in PDAC cells requires CtBP2

Having shown CtBP2-dependent ErbB3 regulation in PDAC cells and tumors, we next explored the potential functional significance of this pathway, including the signaling partners of ErbB3 and their dependence on CtBP activity. ErbB3 signals only when heterodimerized with other EGFR superfamily members that encode active tyrosine kinase domains, such as EGFR, ErbB2, or ErbB4 [11]. Hence, we examined EGFR, Erbb2, and Erbb4 expression in CKP and CKP/KO cells and surprisingly found a significant decrease in Erbb2, but not EGFR levels, in CKP/KO cells relative to CKP cells (Fig. 3A), while Erbb4 was not detectably expressed (data not shown). We found similar results after stable knockdown of CtBP2 in HPAC cells, with a drastic reduction in *ErbB2/3* mRNA and protein levels in shCtBP2-expressing vs. shCtrl-expressing HPAC cells (Fig. 3B). These results align with human tumor data where ErbB2 and ErbB3 are expressed in synchronous fashion in PDAC [12] and in other cancers, such as breast cancer [13]. In terms of the potential clinical significance of these findings in PDAC patients, interrogation of the TCGA database revealed significantly higher levels of both *ErbB2* and *ErbB3* mRNA in PDAC tumors compared to adjacent normal pancreatic tissue (Fig. S4A and S4B), and importantly, survival after PDAC diagnosis is inversely correlated with *ErbB3* mRNA expression level [14]. Indeed, shRNA knockdown of ErbB3 in HPAC cells severely attenuated the rate of cell growth in culture as compared to HPAC cells expressing a control shRNA (Fig. 3C).

Next, we interrogated the functional significance of CtBP2 regulation of ErbB2/3 for oncogenic downstream signal transduction, as ErbB2/ErbB3 heterodimers actively signal through both the PI3K/Akt and MAP Kinase (MAPK) pathways [10]. Serum-starved CKP and CKP/KO cells were treated with the ErbB3-specific ligand NRG-1 [15] at increasing doses (0, 0.25, 0.5, 1 ng/ml) for 15 min and cell lysates analyzed for Akt/MAPK signaling by immunoblotting for total and activated phosphorylated (p-) forms of Erbb2, Erbb3, Akt, and Erk1/2. In CKP cells, NRG-1 stimulation activated Erbb2/Erbb3/Akt/MAPK signaling as evidenced by dose-dependent induction of phosphorylated species of Erbb2, Erbb3, Akt, and Erk1/2 (Fig. 3D). However, CKP/KO cells showed little or no induction of Erbb2 or Erbb3 phosphorylation upon treatment with NRG-1 due to low total Erbb2/3 protein levels, and consistent with decreased Erbb2/3 signaling, downstream Akt and Erk1/2 phosphorylation was also absent. These results support Ctbp2 as a critical regulator of Erbb2/Erbb3/Akt/MAPK signaling in PDAC cells.

### Sensitivity to inhibitors of EGFR-family tyrosine kinases is regulated by the CtBP2/ErbB3 axis

**Fig. 4:**
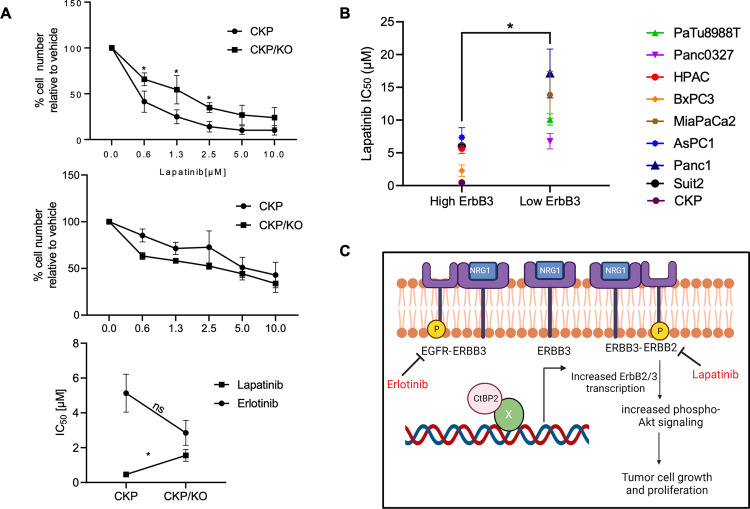
TKI sensitivity of PDAC cells correlates with CtBP2 and ErbB3 expression. **A** CKP or CKP/KO cells were treated with indicated concentrations of lapatinib (top panel) or erlotinib (middle panel) over 72 h, and plates were stained with crystal violet, and the relative cell number at each concentration was determined by extraction of the crystal violet in each well with glacial acetic acid and determination of absorbance at 590 nm. (bottom panel) IC_50_ values for CKP and CKP/KO cells treated with lapatinib or erlotinib. **B** Lapatinib IC_50_ values were determined for the indicated human PDAC cell lines using the crystal violet assay as described in (**A**). **C** Proposed CtBP/ErbB2/3 regulatory axis in PDAC. CtBP proteins partner with unknown DNA binding transcriptional factors regulating the gene expression of ErbB2/3 in PDAC contributing to tumor growth and metastasis. Deletion or depletion of CtBP2 downregulates ErbB2/3 levels leading to decreased tumor growth. The active ErbB3 signaling complexes are heterodimers with either EGFR or ErbB2 in PDAC cells. The dependency of lapatinib cell inhibition on ErbB3 expression suggests that ErbB2/ErbB3 heterodimers are functionally active signaling complexes for PDAC cell growth or survival in both mouse and human PDAC models (Figure was created with Biorender.com; Toronto, CA). (*n* = 3 independent experiments). *p < 0.05. *p* Values were obtained by performing paired (**A**) or unpaired (**B**) *t*-test between the two groups. Error bars indicate ±1.0 SD. *Methods:* (TKI IC_50_ determinations) Human PDAC cell lines Panc-1, HPAC, MiaPaCa2, AsPC1, Panc0327, and BxPC3 were obtained from ATCC. The SUIT2 and PaTu8988T PDAC cell lines were the generous gift of T. Donahue (University of California, Los Angeles) and were verified by STR analysis (University of Arizona Genetics Core), and all cell lines used in this experiment were confirmed to be mycoplasma-free using a PCR-based assay as described in Fig. 1 Methods. The indicated mouse or human PDAC cell lines were seeded in 6 well plates (1×10^4^/well) and treated with indicated lapatinib or erlotinib (Cayman Chemicals, Ann Arbor, MI, USA) concentrations for 72 h, followed by staining with 0.5% (w/v) crystal violet. For quantitation, the stain was dissolved in 10% glacial acetic acid and the absorbance measured at 590 nm. IC_50_ values were determined using GraphPad Prism v8.1 software (GraphPad Software, Boston, MA, USA).

To determine if physiologic EGFR-family signaling in PDAC cells that express ErbB3 could be pharmacologically targeted for therapeutic benefit, we explored the sensitivity of CKP and CKP/KO cells to inhibitors that target active heterodimeric EGFR/ErbB3 and ErbB2/ErbB3 signaling complexes. For this purpose, we exposed CKP and CKP/KO cells to the EGFR/ErbB2 dually specific tyrosine kinase inhibitor (TKI) lapatinib or the EGFR-specific TKI erlotinib. Of note, erlotinib exhibits little or no cross-inhibition of ErbB2 kinase, while lapatinib inhibits both ErbB2 and EGFR/ErbB1 kinases [16, 17]. We found that CKP cells were ~3-fold more sensitive to lapatinib than CKP/KO cells (IC_50_s 0.4 µM and 1.4 µM, respectively; *p* < 0.05), which could be explained by the decreased Erbb2/3 expression and signaling in CKP/KO cells when compared to CKP cells (Fig. 4A, upper and lower panels). We further tested lapatinib sensitivity across additional human PDAC cell lines with differential expression of ErbB3 (Fig. S3), and consistent with results in CKP vs. CKP/KO cells, cells with low or undetectable ErbB3 expression showed significantly higher lapatinib IC_50_ values compared to cells with high ErbB3 expression (Figs. 4B and S3). To determine if inhibition of EGFR/ErbB3 complexes by lapatinib might be contributing to its PDAC cell inhibitory activity, we observed that, unlike lapatinib, the IC_50_ of the EGFR-specific TKI erlotinib was not significantly different between CKP and CKP/KO cells, (IC_50_s = 5.6 and 2.6, respectively; *p* = NS; Fig. 4A, middle and lower panels).

In this study, we have developed a novel orthotopic allograft PDAC mouse model to show that loss of the oncogenic transcription factor Ctbp2 [2] decreases PDAC tumor growth and metastasis. Indeed, ~100% of mice in the CKP cohort demonstrated peritoneal metastases along with malignant ascites, while only ~20% of mice in the CKP/KO cohort demonstrated peritoneal metastases, and none developed ascites. Demonstrating the faithfulness of this model, the phenotype mirrors that observed of allelic loss of *Ctbp2* in CKP transgenic mice, which abrogated the development of ascites and peritoneal metastases that is universal to the CKP model [4]. In contrast to the costly and time-consuming transgenic CKP model, the CKP orthotopic allograft model serves as a tractable PDAC mouse model that will aid study of tumor progression and metastasis mechanisms, as well as potential therapeutic strategies for PDAC. Though our data was generated in immunodeficient mice to focus on the cell autonomous effects of *Ctbp2*, the model also has the significant advantage of allowing study in immunocompetent syngeneic (C57B/6) mice that would facilitate studies of mechanisms of immune escape as well as immunotherapeutic strategies, the lack of which are an enormous unmet need in human PDAC.

Upon mechanistic exploration of how Ctbp2 might be regulating PDAC tumor progression, an unbiased transcriptomic profiling approach revealed that loss of *Ctbp2* significantly downregulated expression of the Erbb3 growth factor receptor, a member of the EGFR family of growth factor receptors, which is expressed in a subset of human PDAC tumors [12, 18] and has been previously linked to adverse clinical outcomes in both PDAC and breast cancer [14, 19]. Validating these RNA-seq results, directed qPCR of orthotopic CKP vs. CKP/KO tumor mRNA revealed striking decreases in mRNA and protein levels of *Erbb3*, as well as its signaling partner *Erbb2*, in tumors lacking *Ctbp2*. Moreover, *Ctbp2* loss abrogated downstream signaling to the growth-promoting Akt/MAPK pathways after engagement of Erbb3 by its native ligand, NRG-1. Consistent with a critical role for ErbB3 signaling in maintaining the PDAC growth phenotype, depletion of ErbB3 dramatically attenuated the growth of human HPAC PDAC cells. Thus, our findings suggest that CtBP2 regulates PDAC growth and metastasis via concerted transcriptional regulation of *ErbB2* and *ErbB3*, and that ErbB3 is critically required to maintain growth of PDAC cells that maintain its expression via active signaling by at least, the Akt/MAPK pathways (Fig. 4C). Notably, this work is the first to address whether native expression of ErbB3 in PDAC cells is actually required to maintain growth capacity, as a prior report suggesting ErbB3 plays an important role in PDAC tumor growth and progression relied on exogenous overexpression of ErbB3 [18].

Our data strongly supports targeting of ErbB2/3 signaling in ErbB3-expressing PDAC, and our work also supports an emerging paradigm in the field of EGFR-family targeted therapeutics, based on recent data showing: (1) Responsiveness of ErbB2(low) tumors to anti-ErbB2 agents [20] as almost all PDAC would be classified as ErbB2(low) [21]; (2) the critical nature of ErbB2/ErbB3 complexes in oncogenic signaling evidenced by the success of pertuzumab that targets ErbB2/3 heterodimerization in breast cancer refractory to trastuzumab [22], and; (3) the improvement in survival seen with dual targeting of ErbB2/3 with trastuzumab and the ErbB2-specific TKI tucatinib [23]. Our data support further investigation of a similar dual targeting strategy in ErbB2(low)/ErbB3(high) PDAC tumors using extracellular domain directed antibodies against ErbB2/3 interaction, such as pertuzumab, or against ErbB3 itself [24, 25], combined with TKI inhibition of the ErbB2 kinase, with lapatinib as we report here, or with the next generation TKI tucatinib, as has shown success in breast cancer [23].

### Supplementary information

Supplemental Material

## Data Availability

The datasets generated during the current study are available in the GEO repository.
