# Supplementary material for: Coordinate transcriptional regulation of ErbB2/3 by C-terminal binding protein 2 signals sensitivity to ErbB2 inhibition in pancreatic adenocarcinoma
Source: Oncogenesis. 2023 Nov 10;12(1):53. doi: 10.1038/s41389-023-00498-8 (PMC10638350; doi:10.1038/s41389-023-00498-8)
Supplement: Supplementary file 1 — Supplemental Material [file 41389_2023_498_MOESM1_ESM.pdf]

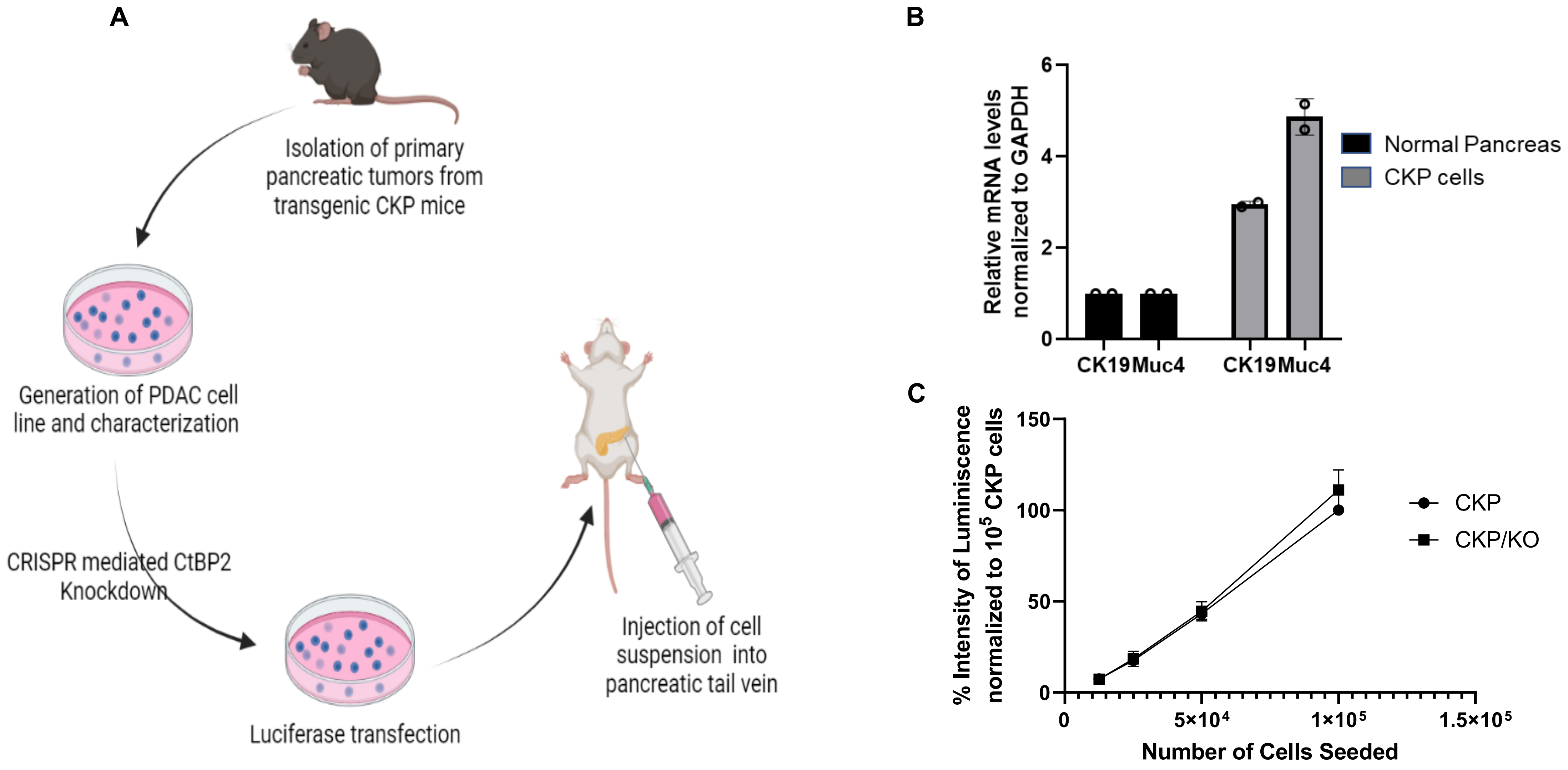

Fig. S1: Development and characterization of the CKP PDAC cell line. (A) Graphic description of the development of orthotopic CKP PDAC model. **(B)** mRNA expression of the PDAC markers *Muc4* and *Krt19* (CK19) was analyzed in CKP cells relative to normal mouse pancreas tissue using qPCR (n=2). **(C)** *In vitro* quantification of luciferase expression in CKP-luc and CKP/KO-luc cells (n=2). Error bars indicate SEM.

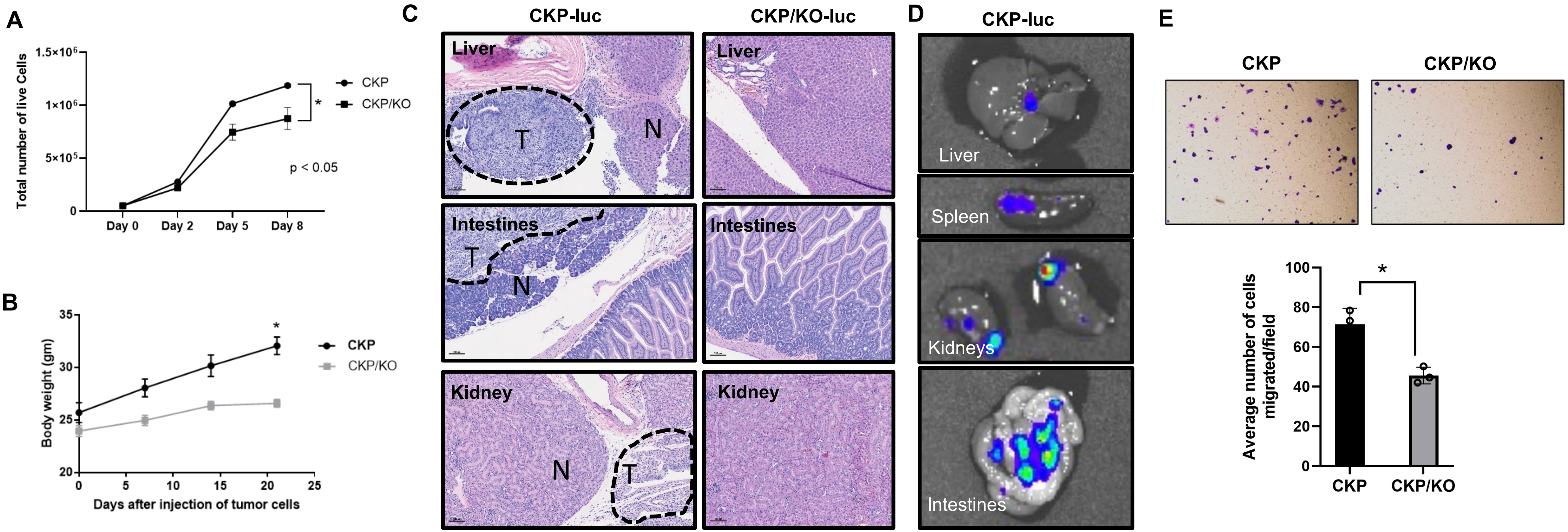

Fig. S2: CtBP2 deletion decreases PDAC growth and metastasis. (A) Cell growth assay of CKP-luc vs. CKP/KO-luc cells. **(B)** Body weight of mice post orthotopic tumor cell injection of CKP-luc and CKP/KO-luc cells. **(C)** Representative H&E staining of tissue from organs at necropsy of mice from **Fig. 1** with CKP-luc or CKP/KO-luc tumors. Metastatic tumor deposits are denoted by dashed lines. T: Tumor ; N: Normal tissue. Scale bars: 100 µm. **(D)** *Ex vivo* bioluminescent imaging of metastatic tumors in organs of mice from **Fig. 1** with CKP orthotopic tumors. **(E)** Trans-well migration assay of CKP vs. CKP/KO cells. (**top panel**) Representative crystal violet images of migrated cells on the transwell membrane. (**bottom panel**) Average number of cells migrated per field from five random fields of the transwell membrane stained with crystal violet (n=3). *$p<0.05$ using paired t-test between the two groups. Error bars indicate +/- 1.0 SD.

<u>*Methods:*</u> Approximately, 4×10^4 CKP or CKP/KO cells were seeded in serum-free RPMI media on a transwell migration insert (Transwell polycarbonate membrane cell culture inserts; Cat. No. 3428, Corning, Corning, NY, USA) placed in a six well culture plate. The inserts were incubated with regular media overnight for better adherence of the cells as specified by the manufacturer, before addition of cell suspension to the inserts. The bottom of the well contained regular RPMI media with 10 % FBS that acts as chemoattractant for the migration of cells. After 24 h, cells were washed with cold PBS and stained with 0.5 % (w/v) crystal violet in 20% methanol. The percentage of cells migrated was analyzed by counting the number of cells stained with crystal violet on the transwell migration membrane from five random microscope fields.

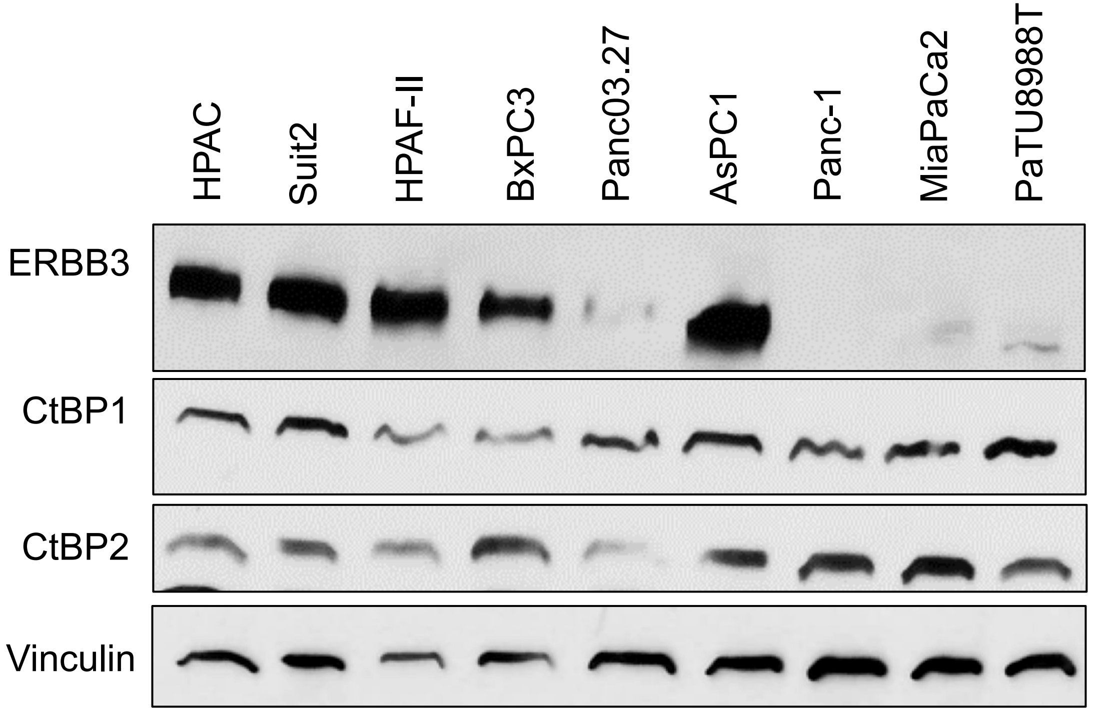

Fig. S3: **CtBP1/2 and ErbB3 protein expression in human PDAC cell lines**. CtBP1/2 and ErbB3 expression levels were determined by immunoblotting lysates from the indicated human PDAC cell lines with vinculin as loading control.

Methods: The HPAF-II PDAC cell line (gift of A. Ivanov, Cleveland Clinic) was verified by STR analysis (University of Arizona Genetics Core) and confirmed to be mycoplasma-free using a PCR-based kit (Abcam). All other PDAC cell lines used in this figure are described in the Methods section of **Fig. 4**. ERBB3 and CtBP2 antibodies and immunoblotting procedure are described in the Methods section of **Fig. 3**. CtBP1 (Cat No: 612042, 1:1000; BD Biosciences) and vinculin (Cat No: 13901, 1:5000; Cell Signaling Technologies) antibodies were immunoblotted according to the procedure in the Methods section of **Fig. 3**.

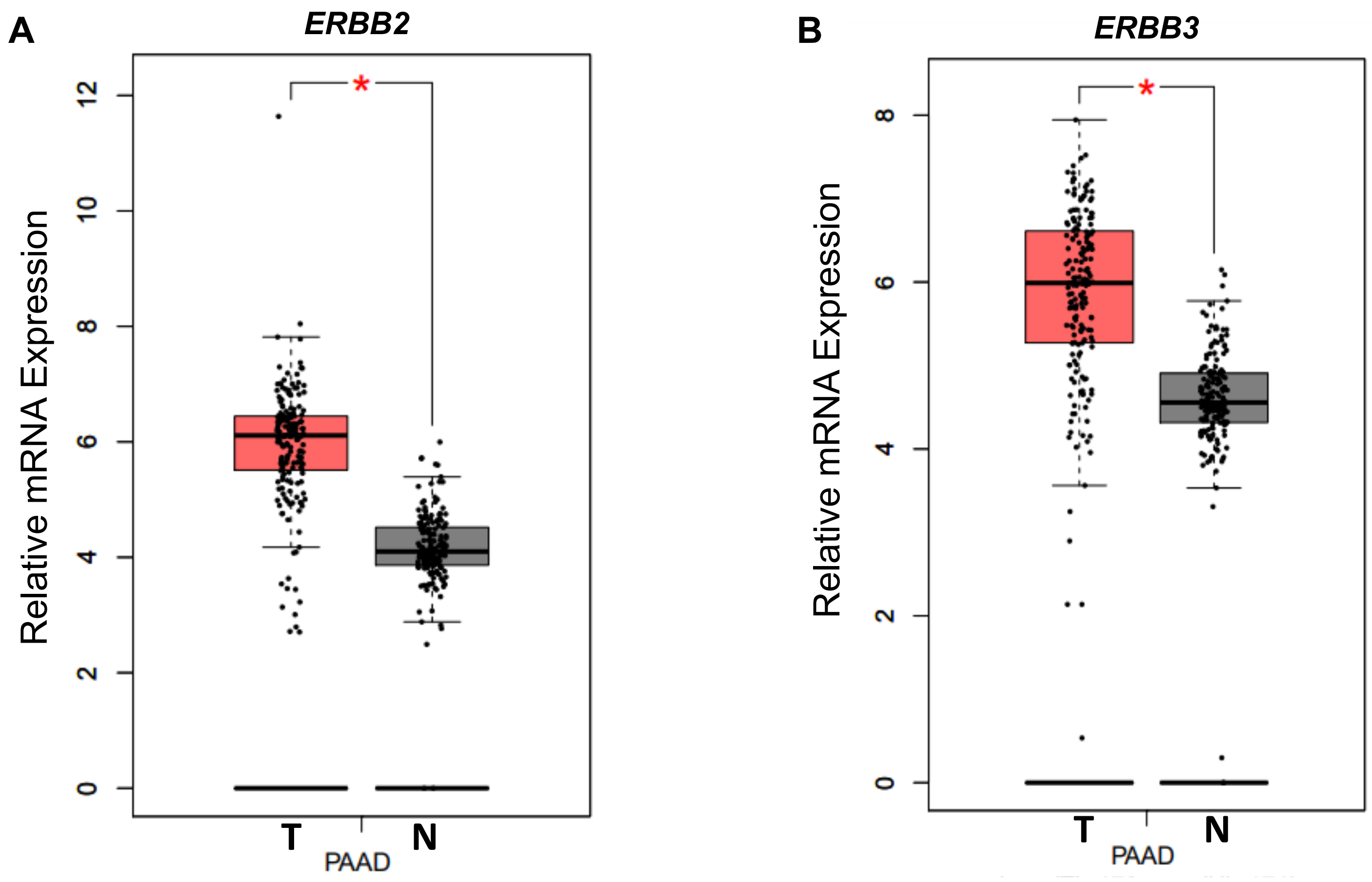

Fig. S4: ***ERBB2/3* expression in human PDAC vs. normal tissue**. Box plots showing relative mRNA expression of **(A)** *ERBB2* and **(B)** *ERBB3* in PDAC tumor vs. pancreatic normal tissues using expression data available in the TCGA (179 pancreatic adenocarcinomas [PAAD] and 4 normal pancreatic tissue specimens) and GTEx (167 normal pancreatic tissue specimens) datasets utilizing the Gene Expression Profiling Interacting Analysis portal (http://gepia.cancer-pku.cn/about.html). T indicates tumor and N indicates normal tissue. * $p < 0.05$ for difference of median relative mRNA expression between tumor and normal tissue for both *ERBB2* and *ERBB3*. Error bars = +/- 1.0 SD.

Organ	CKP	CKP/KO
Liver	5/5	1/5
Intestines	5/5	0/5
Spleen	5/5	1/5
Kidneys	5/5	0/5

Table S1. Number of mice with peritoneal metastases observed on surfaces of the indicated organs after orthotopic pancreatic injection of CKP or CKP/KO cells per the procedure described in the Methods section of **Fig. 1** (n=5/group).

Name	Sequence
CP01	CTGAGAGTGATCGTGCGAATGTTTT
CP02	ATTCGCACGATCACTCTCAGCGGTG
DP331	GCTCTGACCTGTGACTCGTGTCCCTGACAG
DP332	CTGCCTTCTACTCATTCTCCTGTTCACTGAGCATGGC

Table S2: sgRNA-encoding complimentary oligonucleotides targeting exon 3 of *Ctbp2* (CP01/02) and forward/reverse flanking PCR primers used for validation of *Ctbp2* exon 3 homozygous deletion (DP331/332).

Gene	Species	Forward primer	Reverse primer
18S	Mouse	5’-GTAACCCGTTGAACCCCATT-3’	5’-CCATCCAATCGGTAGTAGCG-3’
Erbb3	Mouse	5'-TCTGCATTAAAGTCATCGAGGAC-3'	5'-CAGCCGTACAATGTGGGCAT-3'
Erbb2	Mouse	5’-GAGACAGAGCTAAGGAAGCTGA-3'	5'-ACGGGGATTTTCACGTTCTCC-3'
Ctbp2	Mouse	5’-GGGATAGAACGATCTCTGGGC-3’	5’-AGTGCAAGGAGACGCAGTC-3’
Krt19	Mouse	5’-ACCCTCCCGAGATTACAACC-3’	5’-CAAGGCGTGTTCTGTCTCAA-3’
Muc4	Mouse	5’-GAGGGCTACTGTCACAATGGAGGC-3’	5’-AGGGTTCCGAAGAGGATCCCGTAG-3’
18S	Human	5’-CGCCGCTAGAGGTGAAATTC-3’	5’-TGGCAAATGCTTTCGCTCTG-3’
ERBB3	Human	5'-GGTGATGGGGAACCTTGAGAT-3'	5'-GGTGATGGGGAACCTTGCGAT-3'
ERBB2	Human	5'-TGCAGGGAAACCTGGAACTC-3'	5'-ACAGGGGTGGTATTGTTCAGC-3'
CTBP2	Human	5’-ATCCACGAGAAGGTTCTAAACGA-3'	5’-CCGCACGATCACTCTCAGG-3'

Table S3: qPCR primer pairs used to analyze mRNA expression of *Erbb2/ERBB2*, *Erbb3/ERBB3*, *Ctbp2/CTBP2*, *Krt19*, and *Muc4* genes. Mouse or human *18S* primer pairs were used for internal standard qPCR reactions.
